# Lithotomy—Again

**Published:** 1842-02

**Authors:** 


### LITHOTOMY—AGAIN.

In our last we mentioned the operation for Lithotomy, by Professor Gross, nine days before the issuing of the number. We have now the pleasure to state, that the little patient was sent home, entirely restored, in three weeks. This day (Feb. 1) the Professor operated on another child, three years old. It was done before the class, without accident, and the little patient was sent away, apparently free from all danger. The calculus was small. The child a native of Louisville. D.

